# A Probabilistic Atlas of Diffuse WHO Grade II Glioma Locations in the Brain

**DOI:** 10.1371/journal.pone.0144200

**Published:** 2016-01-11

**Authors:** Sarah Parisot, Amélie Darlix, Cédric Baumann, Sonia Zouaoui, Yordanka Yordanova, Marie Blonski, Valérie Rigau, Stéphane Chemouny, Luc Taillandier, Luc Bauchet, Hugues Duffau, Nikos Paragios

**Affiliations:** 1 Center for Visual Computing, Ecole Centrale Paris, Chatenay Malabry, France; 2 INRIA, Galen Team, Saclay-Ile-de-France Center, Chatenay Malabry, France; 3 Intrasense SAS, Montpellier, France; 4 INSERM U1051, Montpellier Neurosciences Institute, University Hospital, Montpellier, France; 5 Department of Medical Oncology, Institut Régional du Cancer de Montpellier - Val d’Aurelle, Montpellier, France; 6 Department of Clinical Epidemiology and Evaluation, University Hospital, Nancy, France; 7 Department of Epidemiology, Groupe de Neuro-Oncologie du Languedoc-Roussillon, Registre des Tumeurs de l’Hérault, Institut Régional du Cancer de Montpellier - Val d’Aurelle, Montpellier, France; 8 Department of Neurosurgery, HIA Val de Grâce, Paris, France; 9 Neuro-oncology Unit, Department of Neurology, University Hospital, Hôpital Central, Nancy, France; 10 Department of Pathology, University Hospital, Hôpital Gui de Chauliac, Montpellier, France; 11 Department of Neurosurgery, University Hospital, Hôpital Gui de Chauliac, Montpellier, France; University of Pécs Medical School, HUNGARY

## Abstract

Diffuse WHO grade II gliomas are diffusively infiltrative brain tumors characterized by an unavoidable anaplastic transformation. Their management is strongly dependent on their location in the brain due to interactions with functional regions and potential differences in molecular biology. In this paper, we present the construction of a probabilistic atlas mapping the preferential locations of diffuse WHO grade II gliomas in the brain. This is carried out through a sparse graph whose nodes correspond to clusters of tumors clustered together based on their spatial proximity. The interest of such an atlas is illustrated via two applications. The first one correlates tumor location with the patient’s age via a statistical analysis, highlighting the interest of the atlas for studying the origins and behavior of the tumors. The second exploits the fact that the tumors have preferential locations for automatic segmentation. Through a coupled decomposed Markov Random Field model, the atlas guides the segmentation process, and characterizes which preferential location the tumor belongs to and consequently which behavior it could be associated to. Leave-one-out cross validation experiments on a large database highlight the robustness of the graph, and yield promising segmentation results.

## Introduction

Diffuse WHO grade II gliomas (DLGG) are pre-malignant brain tumors characterized by a continuous growth, a migration along the white matter tracts and an unavoidable anaplastic transformation [1]. They affect people in their thirties or forties [2–4]. Two types of tumors are observed depending on their growth speed and the age at the time of diagnosis [5]. An improved knowledge on their natural history has led to shift from a “wait and see” policy to more aggressive therapeutic strategies, with early surgery as the first option, in order to delay malignant transformation while preserving the quality of life of the patients [6, 7].

The management of these tumors is highly dependent on their location. First, several studies have suggested a variability in terms of molecular biology among DLGG with regards to the tumor location [8–11], with a higher rate of 1p deletion in the anterior part of the brain (in particular in the frontal lobe) [8] and a lower rate in the insula [9], or the absence of IDH1 mutation within the insula [10] and its presence for tumors located within the frontal lobe [11]. These differences among DLGGs regarding molecular abnormalities could be one of the explanations to the variability in terms of date of occurrence, growth speed and thus, age at the time of the firsts symptoms or at diagnosis. Second, the dynamical interactions between DLGG and the brain may also vary depending on the eloquence of the areas in which the tumor is located. Indeed, it was shown that slow-growing DLGG might induce cerebral plasticity, explaining the absence of neurological deficit for most patients despite a voluminous tumor, even in the so-called functional regions [12, 13]. Nonetheless, a recent atlas of resectability of DLGG demonstrated that some cerebral areas had low compensatory abilities, constituting a “minimal common brain” among patients [14]. Consequently, the extent of the surgical resection (and thus the median survival) is correlated with the glioma’s location, with a better tumor removal in non-eloquent areas rather than in eloquent ones and in compensable regions rather than non compensable ones [15, 16]. Finally, prognosis can differ according to tumor location. In a recently published series on more than 1000 DLGG patients, frontal locations were associated with a better prognosis compared to other locations, and this could not be entirely explained by the possibility of better resection [4].

Interestingly, it has been suggested that DLGG have preferential locations within the brain, with 82.6% of them located within functional areas in a study analysing 132 DLGG [17]. The supplementary motor area and the insular lobe seem to be their most frequent locations [4, 17, 18]. Nonetheless, to the best of our knowledge, there is no probabilistic map of DLGG locations available in the literature.

The aim of this paper is the introduction of a probabilistic atlas computed on a homogeneous series of patients with a DLGG. The idea is to produce a statistical and compact representation of the tumors’ preferential locations in the brain to facilitate subsequent location-dependent analyses. We will achieve this using binary maps indicating the positions of the tumors obtained by manual segmentation of a series of MRI images. Those maps are registered in the same reference coordinates. A statistical measure evaluates the tumors’ relative position and proximity, and enables to construct a complete graph where each node is a tumor and the arcs’ strength corresponds to the proximity measure. We then aim at reducing the graph’s size to a handful representative nodes situated in the densest areas (i.e. areas where tumors preferentially appear) using unsupervised clustering with unknown number of populations. The quality of the clustering is evaluated using conventional cluster validation methods and cross validation. The interest with regards to a better understanding of the origins of DLGG and their interaction with the central nervous system will be discussed on the basis of this original atlas, as well as possible clinical applications allowing the optimization of the therapeutic management.

The potential and clinical relevance of this atlas are illustrated through two different applications. The first one highlights the interest of location specific studies and how they can be facilitated by our atlas through a comparison between tumour location and the patient’s age. We show how the cluster configuration of our atlas allows for simplified location specific statistical analyses and provide preliminary results suggesting a possible link between age and location.

Our second application highlights how this atlas can be integrated in DLGG segmentation methods and improve their performance by exploiting the knowledge of preferential locations. Knowing the size and extent of the tumor is of key importance for follow up and therapy planning. Currently, the size of a DLGG is approximated by using manual segmentation, a time consuming and tedious task subject to inter expert variability. Automatic tumor segmentation is a difficult task, due to the tumors’ heterogeneous shapes and appearances, overlapping intensities with the surrounding healthy tissue and fuzzy boundaries.

A popular approach for their segmentation is to rely on learning statistical classifiers to separate tumor voxels from healthy voxels. The Support Vector Machines (SVM) classifiers have been extensively studied for this purpose [19–21]. However, this kind of methods have encountered limited success due to the underlying assumption that all voxels are independent and identically distributed (i.i.d), meaning that all voxels are segmented independently from their neighbors. Neighborhood dependent features or morphological filters have been considered to introduce spatial information with limited success. More recently, methods have modeled the spatial dependencies via the use of random fields [22–25]. In this setting, a learned classifier is coupled with a regularization term that penalizes segmentation discontinuities on a defined local neighborhood. The main drawback of those methods is that they encode the spatial dependencies in a local manner, lacking global information on the brain structures’ boundaries. Gering et al. [26] adopted a multilayer MRF approach where an intensity based voxel wise segmentation is progressively refined by incorporating high-level neighboring information such as the distance between the different brain structures. The tumor is detected as a deviation to healthy voxels intensity. User interaction is required for initialization. Corso et al. [27] introduce more global information by coupling Gaussian Mixture Model classification with a multi level graph structure, where the edges of the graph have an affinity that characterizes the similarity between the neighboring nodes.

Atlas-based segmentation methods are endowed with global properties, based on a healthy brain’s expected structures. Kaus et al. [28] alternate statistical classification based on intensity difference with registration of the data with an anatomical atlas, where the tumor voxels are reclassified as healthy. This assumes strong homogeneity in the tumor appearance. Prastawa et al., Moon et al. [29, 30] use a registered probabilistic atlas in which probabilities for tumor and edema are encoded as prior information, based on contrast enhancement. Spatial and geometric constraints are added to avoid false detections. In [31, 32] the tumors are detected as outliers with respect to the normal brain tissue characteristics of a registered healthy atlas. Spatial constraints are modeled via Markov Random Fields [31] or level sets [32]. In [33], we proposed a MRF based coupled tumor segmentation and registration framework where atlas based prior knowledge is introduced through the registration task.

Our method combines local and global informations. Tumor detection is based on a learned classifier and spatial constraints are enforced using a Markov Random Field model, while the statistical atlas of uneven repartition in the brain adds global prior information on the most probable location of tumor voxels. The method is tested on a challenging clinical database with variable quality and poor resolution, and yields promising results that demonstrate the clinical potential of the atlas.

The remainder of this paper is organized as follows: section 2 describes the methodology towards mapping DLGG’s preferential locations in the brain and its application for tumor segmentation. Experimental validation and obtained results are part of section 3 while discussion and perspectives conclude the paper.

## Materials and Methods

### Database Construction and Preprocessing

We conducted a retrospective study at a single institution on a series of 210 DLGG patients. This study was reviewed and approved by the Institutional Review Board (Institut Regional du Cancer de Montpellier - Val d’Aurelle, ID number ICM-URC-2015/35). The patient information was anonymized and de-identified prior to analysis. Patients aged over 18 at diagnosis were included if they fulfilled the following criteria: surgery for a DLGG performed by one of the 3 neurosurgeons of the neuro-oncology unit (HD, LB or YY) between January 1st, 2006 and August 30th, 2012; FLAIR weighted MRI images performed before any oncological treatment (chemotherapy or radiation therapy) available and exploitable. Exclusion criteria were as follows: no histological diagnosis available; low-grade gliomatosis cerebri; tumor volume over 150 *cm*^3^ or multicentric; DLGG located in the spinal cord or in the brainstem; delay >3 years between the first MRI performed (at diagnosis) and the first MRI available. The age at diagnosis (*i.e*. at the time of the first MRI performed), the age at the time of the first symptoms and at the time of the first MRI available were collected for each patient. For incidental tumors (n = 16) the age at the time of the first symptoms was considered to be the age at the time of the first MRI.

The MRI performed at diagnosis was collected for each patient. When that MRI was not available, a later MRI could be used if the patient had not yet received any oncological treatment at the time when it was performed (biopsy possible). The OSIRIX^®^ software was used to perform the image manual segmentation on the FLAIR weighted sequences for each patient. Segmentations were performed by HD and AD. A region of interest (ROI) was defined for each patient, determining for each voxel of each slide of the FLAIR sequence if it belongs to the ROI (i.e. to the tumor) or not. The complete database and expert segmentations are available online at http://db-gliomas-gradeii.net/ and on figshare at http://dx.doi.org/10.6084/m9.figshare.1550871.

Due to our patient selection constraints for atlas construction, all images are acquired in a clinical setting and have therefore variable quality (often low resolution) and progression of the disease. This makes all image processing tasks (tumor segmentation, deformable registration) particularly challenging.

Preprocessing involves intensity regularization for the segmentation task. One of the main issues with MRI imaging is that the intensities of the same anatomical tissues can be very different from one image to another, and even within the same image. Many algorithms have been developed to correct the intensity inhomogeneity [34]. We adopt a simple regularization process:

$$\begin{array}{c} I_{reg}=\frac{I-Med(I)}{IQR(I)} \end{array}$$ (1)

where *Med*(*I*) is the median intensity of the image and *IQR*(*I*) is the interquartile range. Those values are computed without taking into account the background pixels.

### Probabilistic Atlas Construction

A High anatomical variability exists between the brains of different individuals. In order to compare the tumors’ positions in the brain, we need to compensate such variability and bring all the MRI images in the same reference coordinate space. To this end, we perform affine registration towards the same reference pose on all the volumes. While deformable registration would yield better correspondences, the presence of the tumor alters the quality of the registration. The most straightforward approach to deformable registration in such a setting, which we used in a previous work on a much smaller database [35], is to mask the pathology while performing the registration. It has been shown that it can lead to important errors in registration [33] and therefore cannot be used reliably on a large database. Existing methods developed specifically for registration in the presence of tumours [33, 36] currently lack reliability and scalability to be used systematically on our large database, notably due to the variable quality of our data. Let us consider *N* images *I*_*i*_(**x**), *i* ∈ [1, …, *N*] featuring a DLGG, and the healthy reference pose *A*(**x**). All images are affinely registered to the reference pose. We call *M*_*i*_(**x**) the corresponding binary tumor segmentation map obtained by manual segmentation, after affine registration.

Registration brings all the tumors (segmentation maps) in the same coordinate system. The next step is to build a graph expressing the statistical relationships between the tumors’ positions. Using an arbitrary distance measure, we can build a complete graph G where the nodes correspond to the tumors and the edges’ strength corresponds to the distance between the tumors. The nodes’ probability of being at the center of a preferential location increases with the amount of tumors being at proximity (node centrality in the network). As a distance measure, we adopt the euclidean distance between the centers of gravity of the tumors, computed as the mean coordinate among all tumor voxels. This measure has the advantage of being independent from the size of the tumor and of being more robust to registration errors than the use of surface based distances. This strongly reduces the impact of using affine registration over deformable registration. We will now consider **d** = *d*(*M*_*i*_, *M*_*j*_), the set of distances between all possible pairs of tumors.

**Fig 1:**
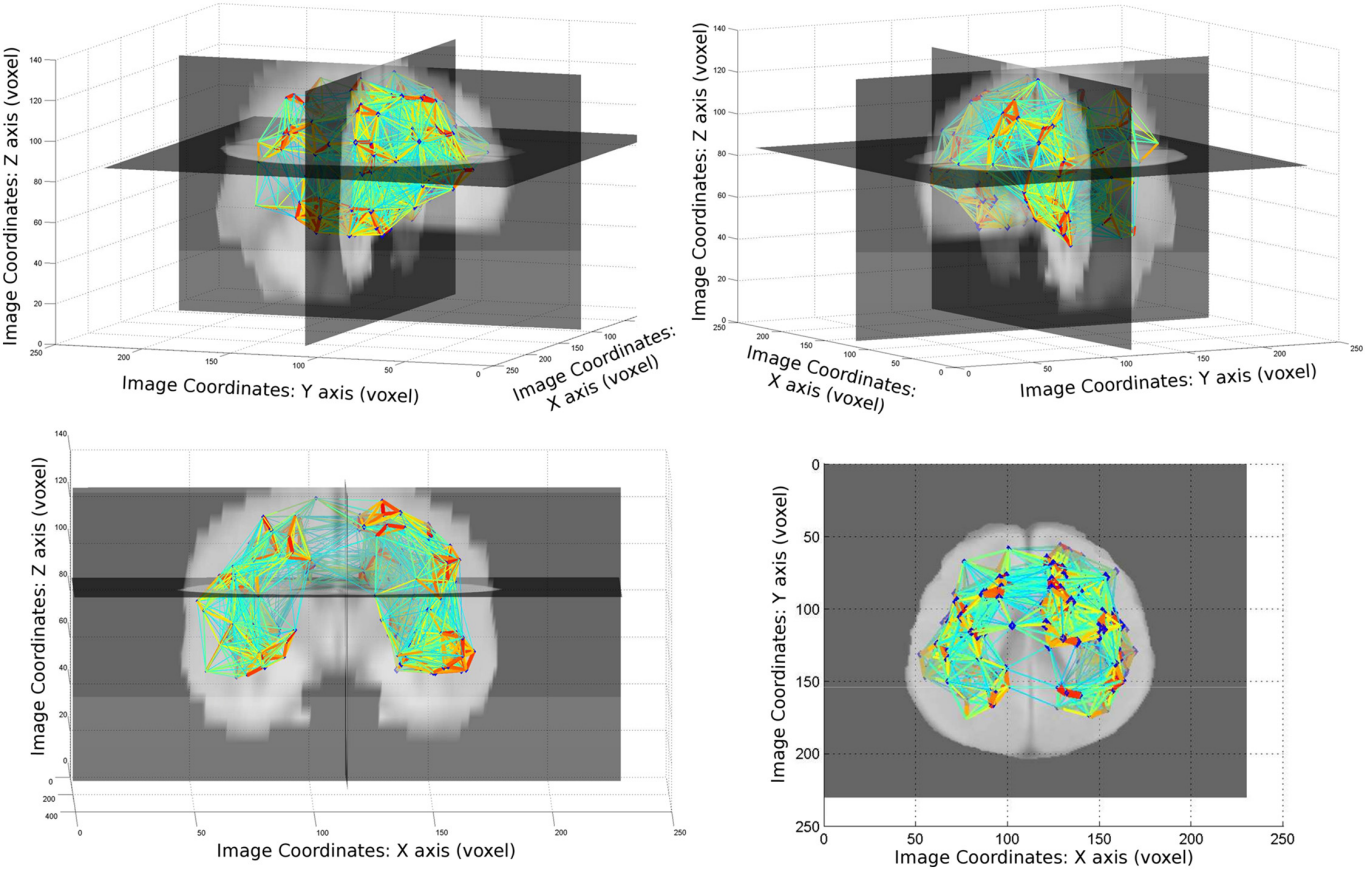
Network representation of the whole data-set before clustering superimposed to the mean registered image. The nodes are located in the center of gravity of the tumors and bigger nodes have bigger network centrality (*i.e*. they are strongly connected to many other nodes). The edges colors and strength represent the distance between nodes (from red and thick (short distance) to blue and thin). For visibility reasons, arcs corresponding to a distance greater than 35 are not displayed.

The complete graph obtained with this distance measure is shown in Fig 1, where the color and thickness of the edges illustrate the distances values (from thick and red (low distance) to thin and blue) while the size of the nodes increases with their centrality.

This graph already suggests some areas with a high density of tumors (red edges) and others where we seldom find tumors. To confirm those observations and identify preferential areas, we aim at reducing the dimensionality of the graph by regrouping close tumors in clusters. If there exists indeed preferential locations, we should obtain compact and well separated clusters. To perform such a clustering, we need to identify (i) the number of clusters *K*, (ii) the centers of the clusters $\bar{c_{1}},\ldots ,\bar{c_{k}}$, (iii) the remaining nodes’ assignments *l*_*i*_, *i* ∈ [1, …, *N*] to the different clusters *c*_1_, …, *c*_*K*_. Popular clustering algorithms such as K-means have two main drawbacks: they are very sensitive to initialization and can easily get trapped in a local minimum, while the number of clusters needs to be predefined. To tackle these issues, we make use of a recently proposed unsupervised clustering method [37] that is able to overcome the aforementioned limitations of conventional methods like K-means. This method automatically determines the optimal number of clusters and is independent from initialization.

$$\begin{array}{c} \min_{k}\min_{c_{1},\ldots ,c_{K}}\min_{\bar{c_{1}},\ldots ,\bar{c_{K}}}\left(\sum_{i=1}^{N}\sum_{i=1}^{K}\delta \left(l_{i},c_{j}\right)d\left(\bar{c_{j}},M_{i}\right)+\alpha \sum_{i=1}^{K}\mu \left(\bar{c_{i}}\right)\right) \end{array}$$ (2)

Where *δ*(.,.) is the Kronecker delta function. First, the nodes assignments should be determined so as to minimize their distance to the closest center (first term). The second term is a penalty introduced in order to avoid the trivial solution of selecting all the nodes as clusters. It is defined as the mean distance value between the considered node and all the remaining nodes:

$$\begin{array}{c} \mu \left(M_{i}\right)=\frac{1}{N}\sum_{j=1}^{N}d\left(M_{i},M_{j}\right) \end{array}$$ (3)

This term enables the nodes with the highest centrality (i.e with the highest amount of close tumors) to be selected as centers. We control the relative importance of the 2 terms using the *α* coefficient. For each *α* value, we evaluate the quality of the clustering using the 3 following indexes:

The Dunn index [38] is very commonly used for cluster evaluation. It compares the biggest distance intra cluster (cluster “diameter”) to the smallest distance inter cluster. The formulation proposed in [38] is very sensitive to noise and outliers as the distances between all nodes are computed. Here, we follow the more robust graph theoretic approach of [39] to define the diameter and distance inter cluster. Each cluster is represented as a Minimum Spanning Tree (MST). Given the set of nodes $V_{j}$ that belong to the cluster *c*_*j*_, a spanning tree is the minimal subgraph to the complete graph that connects all nodes. The weight of the spanning tree corresponds to the sum of the weights of all edges in the tree. The MST $G_{j}^{MST}=\left(V_{j},E_{j}^{MST}\right)$ is the spanning tree of minimal weight. The cluster diameter is then defined as the maximum edge weight among all edges in the MST graph.

$$\begin{array}{c} D=\min_{i\in [1:K]}\left\{\min_{j\in [1:K],j\neq i}\left\{\frac{d(\bar{c_{i}},\bar{c_{j}})}{\max_{k,k^{\prime }\in E_{j}^{MST}}d\left(M_{k},M_{k^{\prime }}\right)}\right\}\right\} \end{array}$$ (4)

A high Dunn index corresponds to compact and well separated clusters (high *d*_*inter*_ and low *d*_*intra*_), i.e. to a good clustering. As a result, we aim at finding a clustering that maximizes the Dunn index.

The Davies-Bouldin [40] index defines a measure of similarity between clusters:

$$\begin{array}{c} R_{i,j}=\frac{\sigma_{i}+\sigma_{j}}{d(\bar{c_{i}},\bar{c_{j}})} \end{array}$$ (5)

and then computes the maximum similarity for each cluster:

$$\begin{array}{c} DB=\frac{1}{k}\sum_{i=1}^{k}\max_{j\in [1:k],j\neq i}R_{i,j} \end{array}$$ (6)

where *σ*_*i*_ is the average distance of all points in cluster *c*_*i*_ to its center. A good clustering corresponds to a low DB index (little similarities between different clusters). Like the Dunn index, DB identifies compact and well separated clusters.

Last, we compute the Silhouette index [41]. It computes for each node a score of confidence with respect to its cluster assignation.

$$\begin{array}{c} s\left(M_{i}\right)=\frac{b\left(M_{i}\right)-a\left(M_{i}\right))}{\max(a\left(M_{i}\right),b\left(M_{i}\right))} \end{array}$$ (7)

*a*(*M*_*i*_) is the average distance between *M*_*i*_ and all the remaining elements assigned to the same cluster while *b*(*M*_*i*_) is the average distance between *M*_*i*_ and all the elements in the closest cluster. The Silhouette index takes values between -1 and 1. If the value is close to 1, the node has been assigned correctly. A value close to zero suggests that the node is equally far away from 2 clusters, while a silhouette index close to -1 infers that the node has been misclassified. To evaluate the quality of the clustering, we compute the global silhouette index:

$$\begin{array}{c} GS=\frac{1}{K}\sum_{j=1}^{K}\frac{1}{n_{j}}\sum_{i=1}^{n_{j}}s\left(M_{i}\right) \end{array}$$ (8)

The highest GS corresponds to the best clustering, where the individual Silhouette indexes are closest to 1.

We select the clustering result (i.e. *α* value) that corresponds to the best indexes value.

### Atlas Application: Tumor Segmentation and Characterization with Position Priors

The probabilistic atlas constructed through clustering provides interesting insight on the locations where tumors are likely to appear and on the tumors’ location dependent properties. Considering a new image I featuring a tumor, assigning it to a cluster could enable to predict its future behavior and spatial repartition based on the cluster properties as well as provide powerful spatial prior information for automatic segmentation.

In this section, we consider that the atlas has been constructed as a *K* clusters graph. For each cluster *c*_*i*_, we now consider the spatial extension of all tumors within this cluster through their associated binary segmentation maps. We build a distribution map as *P*(**x**|*c*_*i*_), *i* ∈ [1: *K*] describing the conditional probability of tumors appearances depending on their location (with respect to the reference frame coordinate system). This can be done simply by counting the number of tumors in the corresponding cluster appearing at each voxel voxel. Considering there are *N* tumors in cluster *c*_*i*_, we defined *P*(**x**|*c*_*i*_) as:

$$\begin{array}{c} P\left(x|c_{i}\right)=\frac{1}{N}\sum_{j\in [1:N]}M_{j}\left(x\right) \end{array}$$ (9)

**Fig 2:**
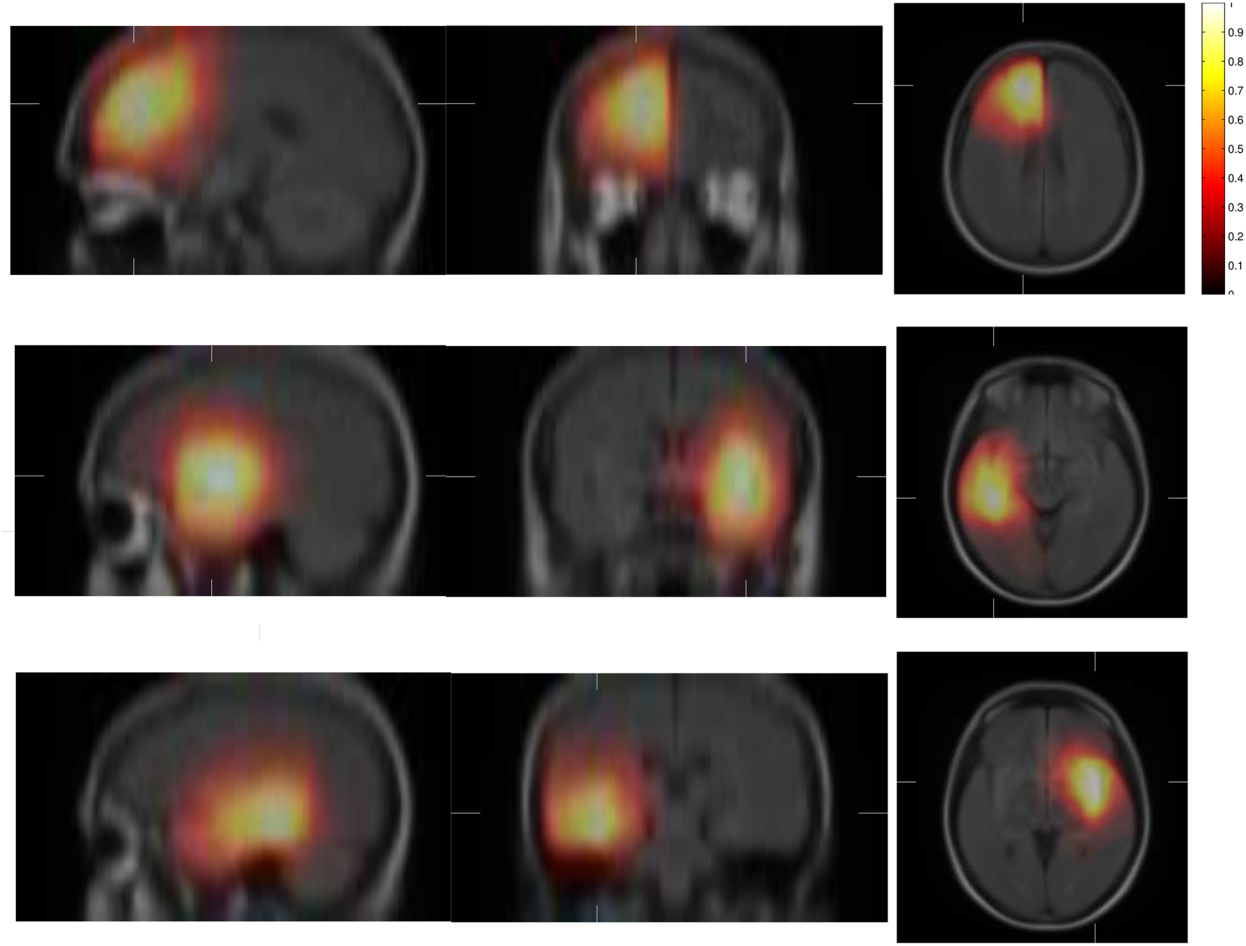
Examples of cluster probability maps describing the spatial repartition of tumors in the cluster. The maps are superimposed to the mean registered image.

To compensate for the possible small amount of tumors per cluster, all probability maps are smoothed using a Gaussian filter. Examples of such probability maps are shown in Fig 2.

We formulate the problem of segmentation and characterization as a labeling problem where we seek to assign a characterization label $\theta_{x}\in C=\left\{c_{1},...,c_{k}\right\}$ and a segmentation label $\omega_{x}\in S=\left\{0,1\right\}$ to each voxel of the image. The characterization label’s role is to determine which preferential location the tumor belongs to by assigning it to one of the sparse graph’s clusters while the segmentation label aims at separating tumor (*ω*_*x*_ = 1) and background voxels (*ω*_*x*_ = 0). We adopt a Markov Random Field (MRF) model on a graph G=(V,E), where the graph nodes V correspond to the image voxels **x** and the graph’s edges E define a first order neighborhood system N by connecting a node to its 6 immediate neighbors. The optimal labeling **l** = [*θ*, *ω*] is recovered by minimizing the MRF energy:

$$\begin{array}{c} E\left(l\right)=\sum_{x}V_{x}\left(l_{x}\right)+\sum_{x}\sum_{y\in N(x)}V_{x,y}\left(l_{x},l_{y}\right) \end{array}$$ (10)

*V*_*x*_(*l*_*x*_) is the unary cost and describes the likelihood of assigning a specific label to voxel **x**, while *V*_*x*, *y*_(*l*_*x*_, *l*_*y*_) describes the pairwise interactions between two neighbouring voxels. The unary and pairwise costs will be details in the subsequent subsections.

#### Detection

Our first step towards tumor segmentation and characterization is the construction of a classifier that will distinguish tumorous and healthy pixels. We learn such a classifier using the boosting algorithm Gentle Adaboost [42] and a set of features extracted from the image. The training set is made of an ensemble of pairs $\left\{x_{i},y_{i}\right\}\in R^{3}\times \left\{-1,1\right\},i\in \left[1:N\right]$, **x**_**i**_ is an observed voxel from a training volume and *y*_*i*_ is its corresponding label. The idea behind boosting is that a strong classifier can be created from an ensemble of weak classifiers. Each pair is associated to a feature vector *f*(**x**_*i*_) and a weight $D_{i}=\frac{1}{N}$. At each iteration t, a weak classifier *h*_*t*_(**x**_**i**_) is constructed as a decision stump in order to minimize the training error $\sum_{i=1}^{N}D_{i}{\left(h_{t}\left(x_{i}\right)-y_{i}\right)}^{2}$. The weights are then updated as *D*_*i*_ = *D*_*i*_ exp(−*y*_*i*_ *h*_*t*_(**x**_**i**_)), putting the emphasis on misclassified sample. Eventually, the strong classifier is computed as a linear combination of the weak classifiers, yielding a score of confidence *H*(*I*(**x**)) for each location of the image to be segmented given the intensity map.

The features used for classification rely on intensity, texture and symmetry. The main feature differentiating healthy and tumorous tissues is the intensity enhancement of tumor pixels on FLAIR images. We exploit this characteristic by using 9 × 9 × 5 intensity patches centered on the pixel of interest. The underlying idea is also to get information about the neighborhood of the pixels since there exists overlapping intensities between tumor and background. Rotation invariant intensity statistics are also used, as we computed the median, standard deviation and entropy of intensity patches of size *k* × *k* × 3, where *k* = [3, 5, 7], all centered on the pixel of interest. Gabor filters [43] are particularly useful for distinguishing objects of different textures. The filters are used on 2 scales and 10 orientations in order to detect the main structures of the brain (such as the skull). The brain’s hemispheres have the interesting property of being roughly symmetrical. This can be taken advantage of in our case, since the tumor introduces dissymmetry between the 2 hemispheres. Thanks to affine registration, the symmetry plane of all images is roughly equivalent to the approximated reference pose’s Π. For each pixel **x** in the left half of the image, we compute a symmetry measure as follows:

$$\begin{array}{ccc} S(x) & = & \frac{1}{N}\sum_{N_{s}\left(x\right)}I\left(x\right)-\frac{1}{N}\sum_{N_{s}\left(x\right)}I\left(x_{\Pi }\right)\\ S(x_{\Pi }) & = & -S(x) \end{array}$$ (11)

where **x**_Π_ is the symmetric of **x** with respect to Π, $N_{s}\left(x\right)$ is a neighborhood of **x** and N the number of pixels in $N_{s}\left(x\right)$. We use a neighborhood to compensate the fact that the symmetry plane is approximate.

#### Pairwise Potentials

The MRF energy’s pairwise potential acts as smoothing priors, imposing spatial consistency of the labellings. We adopt a classic Potts model, penalizing neighboring nodes that are assigned different labels:

$$\begin{array}{c} V_{x,y}\left(\left[\theta_{x},\omega_{x}\right],\left[\theta_{y},\omega_{y}\right]\right)=\{\begin{array}{cc} 0, & \;\text{if}\;\theta_{x}=\theta_{y}\;\text{and}\;\omega_{x}=\omega_{y}\\ \beta , & \;\text{if}\;\theta_{x}=\theta_{y}\;\text{and}\;\omega_{x}\neq \omega_{y}\\ \infty , & \;\text{otherwise}\; \end{array} \end{array}$$ (12)

where *β* is a constant parameter imposing the strength of the penalization. Based on the hypothesis that there is only one tumor per patient, the characterization label has to be the same for all grid nodes. Setting the penalization to infinity imposes the same characterization label on all nodes.

#### Unary Potentials

The unary potential represents the nodes likelihoods and seeks the most likely label configuration. It is composed of three terms:

$$\begin{array}{c} V_{x}\left(\left[\theta_{x},\omega_{x}\right]\right)=V_{S,x}\left(\omega_{x}\right)+V_{C,x}\left(\theta_{x}\right)+V_{C,S,x}\left(\theta_{x},\omega_{x}\right) \end{array}$$ (13)

The segmentation unary potential *V*_*S*, *x*_(.) seeks the most likely segmentation configuration based on the classification likelihoods recovered from the boosting classification decisions. We follow the approach of [44] and define it as:

$$\begin{array}{c} V_{S,x}\left(\omega_{x}\right)=-w_{x}\log P_{tm}\left(I\left(x\right)\right)-\left(1-w_{x}\right)\log P_{bg}\left(I\left(x\right)\right) \end{array}$$ (14)

where the prior probabilities are computed from the boosting classification score as:

$$\begin{array}{ccc} P_{tm}\left(I\left(x\right)\right) & = & {\left(1+\exp(-2H(I(x)))\right)}^{-1}\\ P_{bg}\left(I\left(x\right)\right) & = & 1-P_{tm}\left(I\left(x\right)\right) \end{array}$$ (15)

The role of the characterization term *V*_*C*, *x*_(.) is to identify the closest cluster the tumor to be segmented belongs to. In order to identify the most likely cluster, we measure the degree of non overlap of pixels with a high boosting classification score *H*(*I*(**x**)) with the center of each cluster.

$$\begin{array}{c} V_{C,x}\left(\theta_{x}\right)=\max\left(0,H_{bin}\left(I\left(x\right)\right)-M_{\theta_{x}}\left(x\right)\right) \end{array}$$ (16)

where *M*_*θ*_*x*__(.) is the binary map associated to the center of the corresponding cluster and *H*_*bin*_(.) is the boosting score converted into a thresholded (with threshold T) binary map:

$$\begin{array}{c} H_{bin}\left(I\left(x\right)\right)=\{\begin{array}{cc} 1, & \;\text{if}\;H(I(x))>T\\ 0, & \;\text{otherwise}\; \end{array} \end{array}$$ (17)

Basically, *V*_*C*, *x*_(.) counts the number of pixels with a boosting score beyond a given value that do not overlap with the center of the cluster. The value of *θ*_*x*_ that minimizes *V*_*C*, *x*_ is the closest cluster (highest overlap with the cluster center).

The coupling term *V*_*C*, *S*, *x*_(.,.), linking the segmentation and characterization, is the key element of our framework. The selected cluster gives information on where the tumor voxels are expected to appear and not to appear, according to the spatial distribution of tumors in the cluster. This term plays the part of a spatial position prior and is defined as:

$$\begin{array}{c} V_{S,C,x}\left(\theta_{x},\omega_{x}\right)=-w_{x}\log P\left(x|\theta_{x}\right)-\left(1-w_{x}\right)\log\left(1-P\left(x|\theta_{x}\right)\right) \end{array}$$ (18)

This term ensures that the segmentation labels are consistent with the location corresponding to the assigned type of tumor: tumor detections that are not in accordance with the expected spatial position are penalized.

The MRF optimization is done using Fast PD [45], an optimization method based on linear programming, which offers a great compromise between speed and performance.

### Second Application: Correlation with Patient’s Age: Statistical Analysis

Patients’ characteristics, including age at the time of the first symptoms and age at MRI diagnosis (*i.e*. age at the time of the first MRI), were described using percentages for categorical variables, and mean, standard deviation, median and range for continuous variables. Ages in each cluster (at the time of the first symptoms and at MRI diagnosis) were described with boxplots. An analysis of variance (ANOVA) and a Bonferroni correction for multiple tests were used to compare the mean ages of each cluster. Statistical analysis was performed using the SAS (Statistical Application System) v9.3 software.

## Results

Two hundred and ten patients were included, among them 97 women (46.2%) and 113 men (53.8%). The median age at the time of the first symptoms, at the time of the first MRI and at the time of the MRI analyzed were 37 years (range 15-70), 37 years (range 17-70) and 37 years (range 17-70), respectively. The mean age at the time of the first symptoms, at the time of the first MRI and at the time of the MRI analyzed were 38 years. A surgical resection of the tumor was performed for 202 patients (96.2%) and a biopsy for 8 of them (3.8%). The median and the mean tumor volumes were 44.7 *cm*^3^ (range 0.1-147.9 *cm*^3^) and 52.5 *cm*^3^, respectively. The median and the mean delays between the first MRI performed (i.e. at diagnosis) and the MRI analyzed were 37 days (range 0-2.8 years) and 115 days, respectively. The initial symptoms were epilepsy in a majority of patients (86.2%), a neurological deficit (speech disturbance, visual deficit or cognitive impairment) in 2.4%, and were unknown in 1.9%. The diagnosis of DLGG was incidental for 9.5% of patients (the brain imaging was performed for another disease or for symptoms that cannot be related to the DLGG).

The image size varied from 144 to 576 in the (x, y) plane and 12 to 213 in the z plane, and the voxel resolution from 0.4 × 0.4 to 1 × 1 *mm*^2^ in the (x, y) plane and 0.9 to 10 mm in the z plane. The tumor size ranged from 0.3 to 180 *cm*^3^. The reference pose used for registration is a 256 × 256 × 24 FLAIR image of a healthy brain, with resolution 0.9 × 0.9 × 5.5.

### Sparse Graph Validation

**Fig 3:**
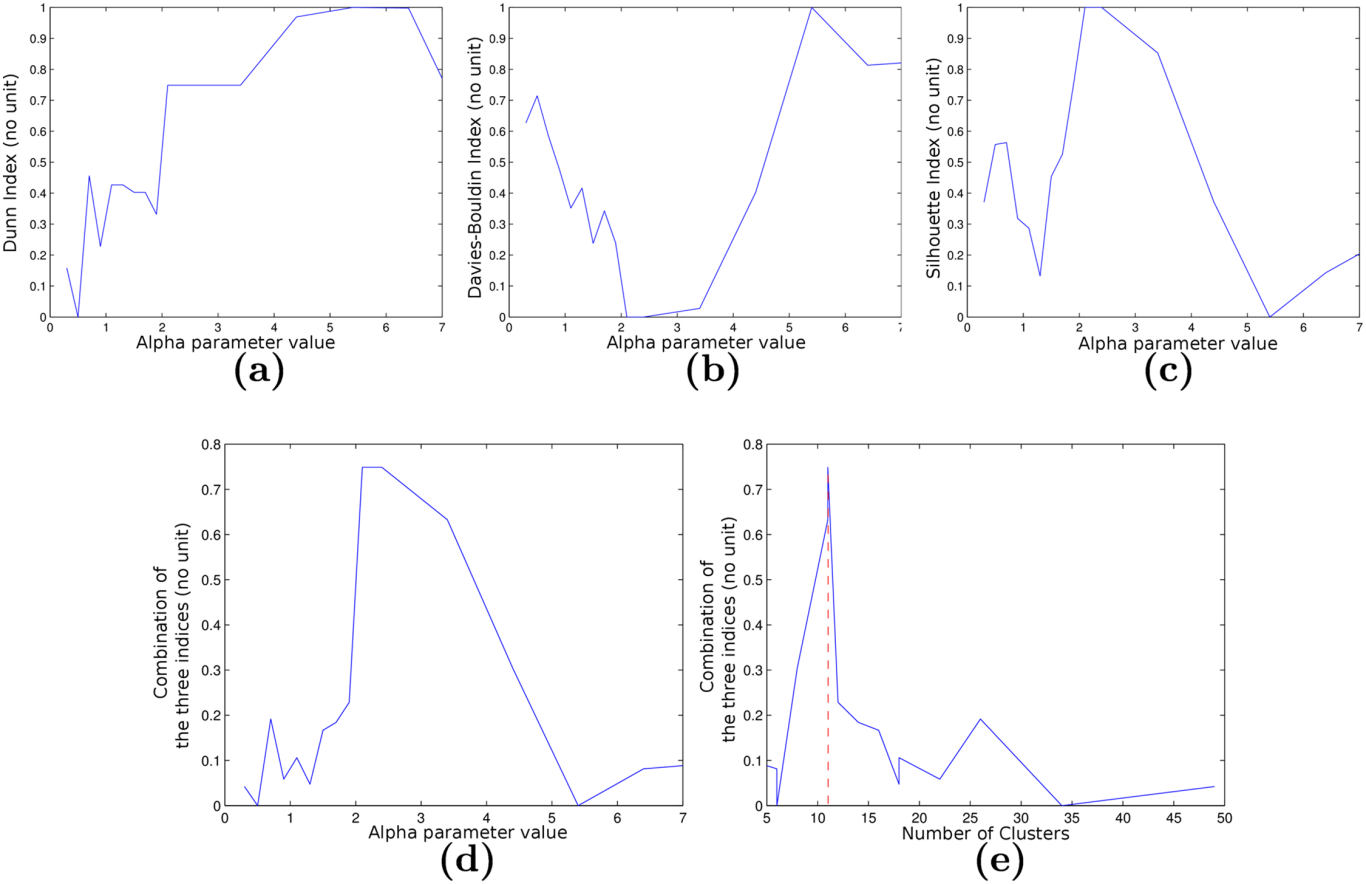
Cluster validity indices with respect to the value of *α* (a, b, c, d) and the number of clusters (e). Dunn index (a), Davies Bouldin index (b), Silhouette index (c) and combined indices (d, e).

**Fig 4:**
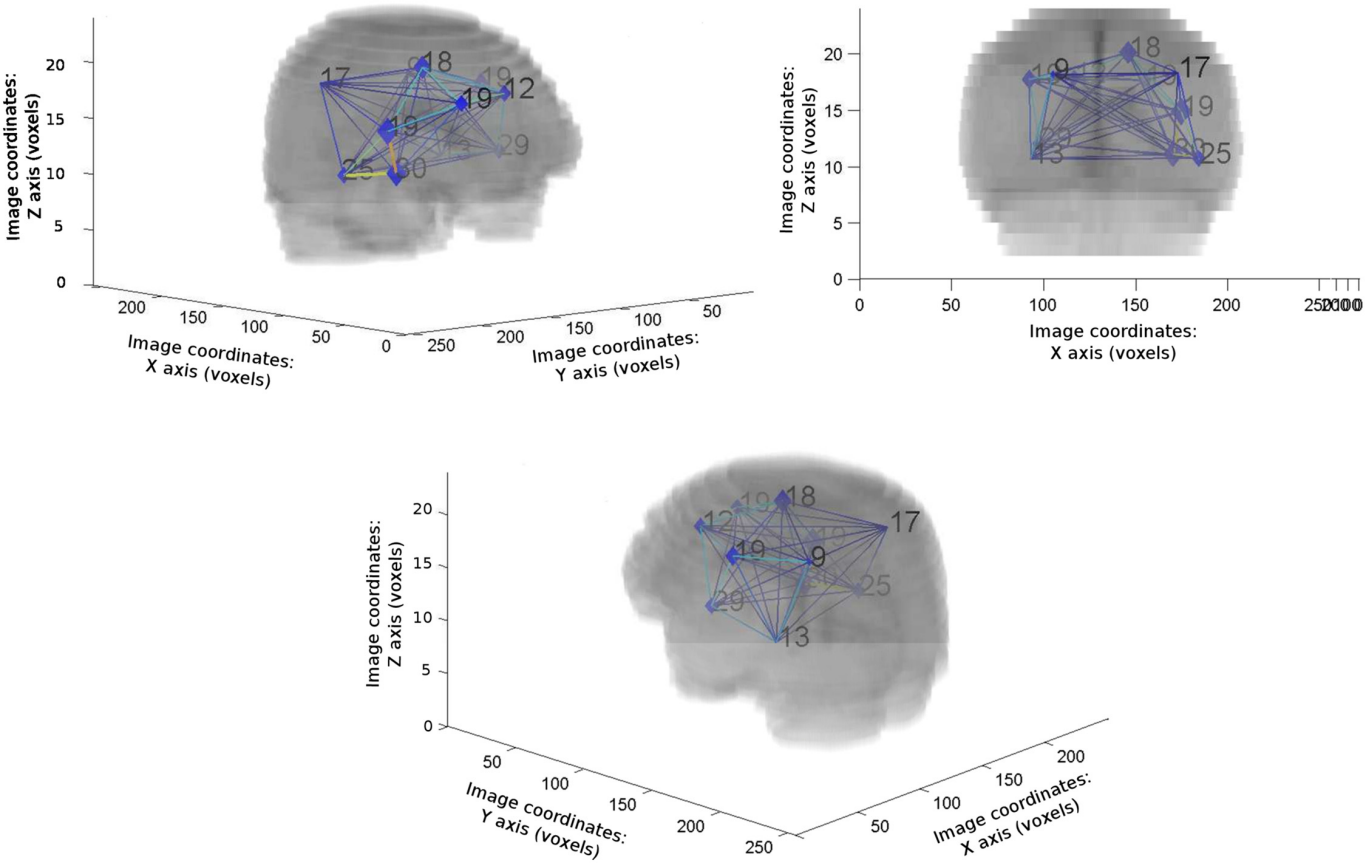
Visualization of the complete clustered graph superimposed to the mean registered image. The numbers correspond to the number of nodes in each cluster.

**Fig 5:**
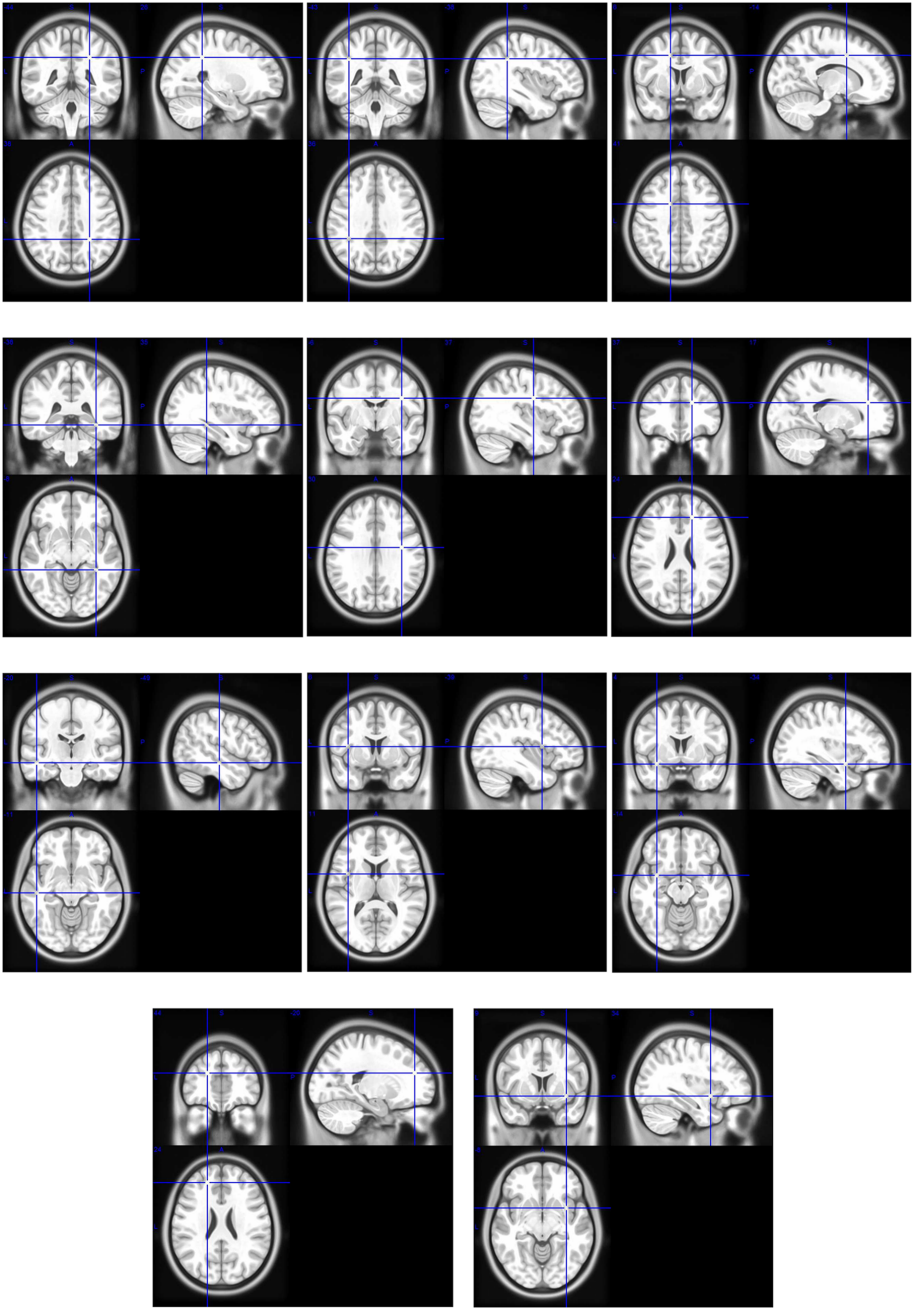
Positions of the cluster centers on the MNI atlas. The clusters are organized in numerical order (from cluster 1 to 11).

In order to select the best clustered graph, we performed clustering of the complete graph for *α* values ranging from 0.3 to 7 producing clustering results involving 5 to 49 clusters. We computed the 3 indexes for all the values *α*. Combining the indexes after normalization as *I*_*c*_ = (*D*.(1 − *DB*).*GS*)^1/3^, we observed a global maximum for *α* = 2.1, corresponding to a 11 clusters graph. Fig 3 shows the different indexes values and their combination, while Fig 4 shows the 11 clusters graph (i.e. the best clustering). We can observe some symmetry in the graph topology. Five out of the 11 clusters are located within the left hemisphere (clusters 1, 4, 5, 6 and 11) while six are located within the right hemisphere (clusters 2, 3, 7, 8, 9 and 10). Clusters 1 and 2 are located in the parietal lobe (left and right parietal lobes, respectively). Clusters 4 and 7 are located within the left and right temporal lobes, respectively, while clusters 8, 9 and 11 are located within the insula. The remaining clusters involve the frontal lobes, with clusters 6 and 10 located in the left and right anterior frontal lobes, respectively, and cluster 3 located within the right prefrontal lobe. The anatomical repartition of clusters is summarized in Table 1 and the positions of the centers of each cluster in the MNI atlas [46, 47] are shown in Fig 5. Positions in the MNI atlas are obtained by non-rigid registration [48] of the FLAIR reference pose to the MNI atlas.

**Table 1:** Anatomical location of the different clusters.

Anatomical location	Number of clusters	Percentage of data
Parietal lobe	2 (symmetric)	12%
Supplementary Motor Area (frontal lobe)	1	9%
Temporal lobe	2 (symmetric)	18%
Frontal lobe	1	9%
Anterior frontal (frontal lobe)	2 (symmetric)	15%
Insula	3 (2 symmetric clusters)	37%

Next, we performed Leave-One-Out Cross Validation to assess the quality of our clustering. For 210 experiments (one per sample), we removed one sample and learned the optimal graph’s topology on the remaining 209 nodes. We then studied the impact of the 210th sample on the structure of the graph and the quality of the clusters. For each of the 210 experiments, we computed the 3 indexes. In most cases (207 samples, 98.5% of the data), the graph was a 11 nodes graph which corresponds to the graph obtained with the full data-set. For 3 samples, the optimal number of clusters was 10, which was due to the fact that they belonged to a loose cluster (center sample or close to center sample) that was merged with its closest neighbor when the sample was removed.

We then studied the robustness of the graph by analyzing the influence of each sample. For each clustering obtained by cross validation, we evaluated the impact of the 210th sample on the structure of the graph and the quality of the clusters. To evaluate how well the clustering represented the data, we tried to assign each removed sample to a cluster. We used three criteria to do so:

1. *The distance from the center of the cluster*. The sample has to be assigned to the closest cluster and should be as close to the corresponding center as the nodes constituting the cluster. To determine if the distance to the cluster center follows the cluster’s distance distribution or if it is an outlier we use rely on the distribution’s quartiles:
  $$\begin{array}{c} d\left(M_{i},\bar{c_{k}}\right)=\min_{j\in [1:K]}d\left(M_{i},\bar{c_{j}}\right)\;\text{and}\;d\left(M_{i},\bar{c_{k}}\right)\leq Q_{3}\left(c_{k}\right)+1.5\left(IQR\left(c_{k}\right)\right) \end{array}$$ (19)
  where *Q*_3_(*c*_*k*_) and *IQR*(*c*_*k*_) are respectively the third quartile and interquartile range of cluster *c*_*k*_’s distance distribution.
2. *The individual Silhouette index*. The global silhouette index is indicative of the quality of the clustering. The individual index tells how well the sample fits in its cluster. Considering the sample belongs to the closest cluster, we compute the corresponding silhouette index. If the index is close to 1, there is no doubt about the fact that the sample belongs to this cluster. A value close to zero does not necessarily mean that the clustering is bad. Indeed, the main drawback of the silhouette is that it can penalize large clusters (large diameter) when compared to smaller ones. To avoid penalizing large clusters, we also compare the individual index to the distribution of silhouette values:
  $$\begin{array}{c} s_{k}\left(M_{i}\right)=\max_{j\in [1:K]}s_{j}\left(M_{i}\right)\;\text{and}\;s_{k}\left(M_{i}\right)\geq Q_{1}\left(c_{k}\right)-1.5\left(IQR\left(c_{k}\right)\right) \end{array}$$ (20)
  where *Q*_1_(*C*_*k*_) and *IQR*(*c*_*k*_) are respectively the third quartile and interquartile range of cluster *c*_*k*_’s silhouette index distribution.
3. The last criterion verifies the consistency between the clusters selected by the first two criteria:
  $$\begin{array}{c} \arg\min_{k}d\left(M_{i},\bar{c_{k}}\right)=\arg\max_{k}s_{k}\left(M_{i}\right) \end{array}$$ (21)

Using those criteria, we manage to classify 88% of the whole data-set without affecting the quality of the cluster (mean distance intra cluster, mean silhouette index).

**Fig 6:**
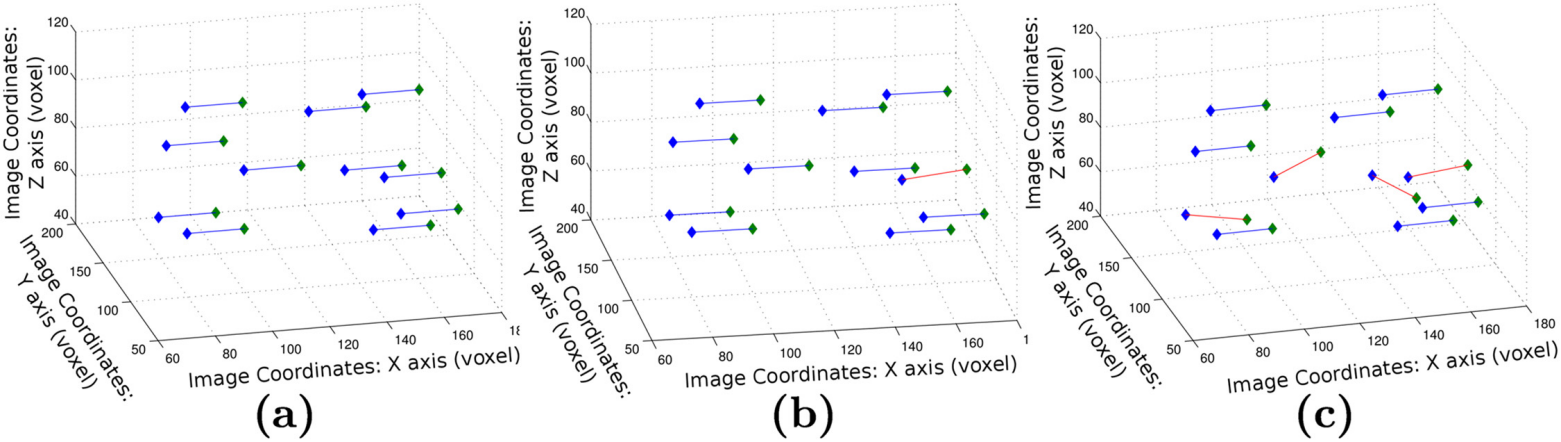
Examples of graph matching results. (a) Complete match, (b, c) Partial match. Positive matches correspond to blue edges and mismatched samples to red edges. The nodes’ locations correspond to the coordinates of the cluster centres of each clustering. The green nodes have been translated along the x axis for visualisation purposes.

We also studied the topology correspondences of the graphs with respect to the original graph *G*_0_. This was only possible for the 207 samples that yielded a 11 clusters graph. Using the algorithm presented in [49], we seek a matching between the nodes and arcs’ strength of the respective graphs. We observed a complete match for 70% of the graphs, and a one node difference for 26%. The remaining graphs differed from 2 nodes (9 cases) and 4 nodes (1 case). In the case of partial matching, the graphs were still very similar as the maximum distance between the different cluster centers was 12.6 which corresponds to the mean intracluster distance of tight clusters. Fig 6 illustrates partial and complete matches, the worst matching case (4 different samples) is illustrated in Fig 6c. We can see that the graphs are still very similar in the case of partial match.

### Segmentation results

The segmentation was evaluated on the 210 volumes through Leave One Out Cross Validation. For each sample, an atlas is constructed using the data’s optimal indexes value and a boosting classifier is learned by randomly selecting 35 volumes among the 209 training volumes. Experiments showed that the potential assigning a cluster to the image (*V*_*clus*_) had to be given a much bigger importance than the others in order to properly label the whole training set to the right cluster (especially if the tumor is small). *T* was set at 1.5, *λ* at 10000, *γ* at 2 and *β* at 1.

We evaluate the quality of our segmentation framework by using the manual segmentations as ground truth. Let us define M the ensemble of pixels labeled as tumor manually, and A the pixels automatically labeled. For each image, we compute the Dice coefficient, False (FP) and True (TP) Positive rates and the Mean Absolute Distance between contours (MAD):

$$\begin{array}{ccc} D & = & \frac{2∥A\cap M∥}{∥A\cup M∥+∥A\cap M∥}\\ TP & = & \frac{∥A\cap M∥}{∥M∥}\\ FP & = & \frac{∥A∥-∥A\cap M∥}{∥A∥}\\ MAD & = & \frac{1}{2}\left(\frac{1}{n_{A}}\sum_{x\in A}d_{min}\left(x,M\right)+\frac{1}{n_{M}}\sum_{x\in M}d_{min}\left(A,x\right)\right) \end{array}$$ (22)

where *n*_*A*_ and *n*_*M*_ are the number of voxels in A and M respectively and *d*_*min*_(*x*, *M*) = inf(*d*(*x*, *y*)|*y* ∈ *M*) is the smallest distance of a point *x* to any point in *M*. The distance *d*(.,.) used here is the euclidean distance.

**Fig 7:**
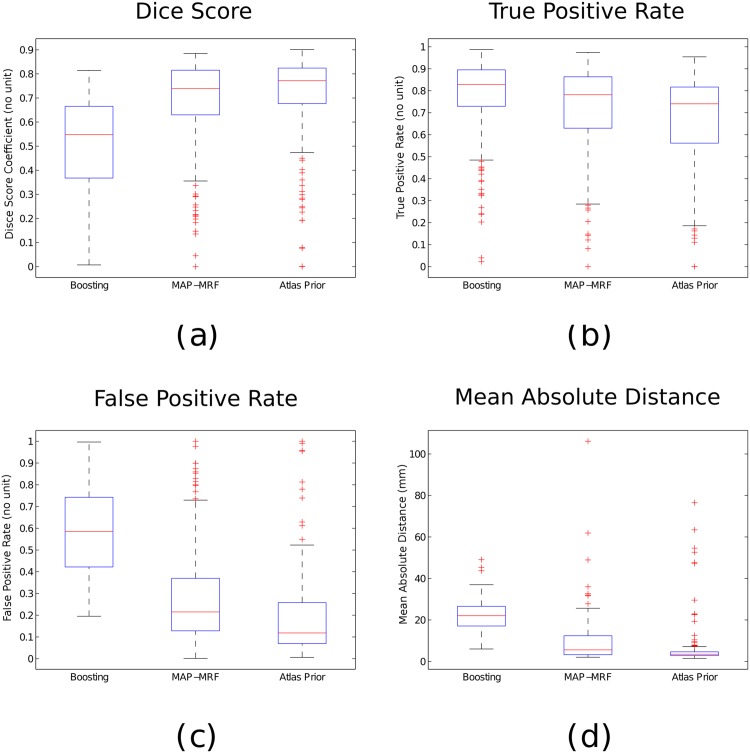
Boxplots of the Dice score (a), True Positive rate (b), False positive rate (c), and MAD score (d) between the automatic and manual tumor segmentation for the three different methods.

**Fig 8:**
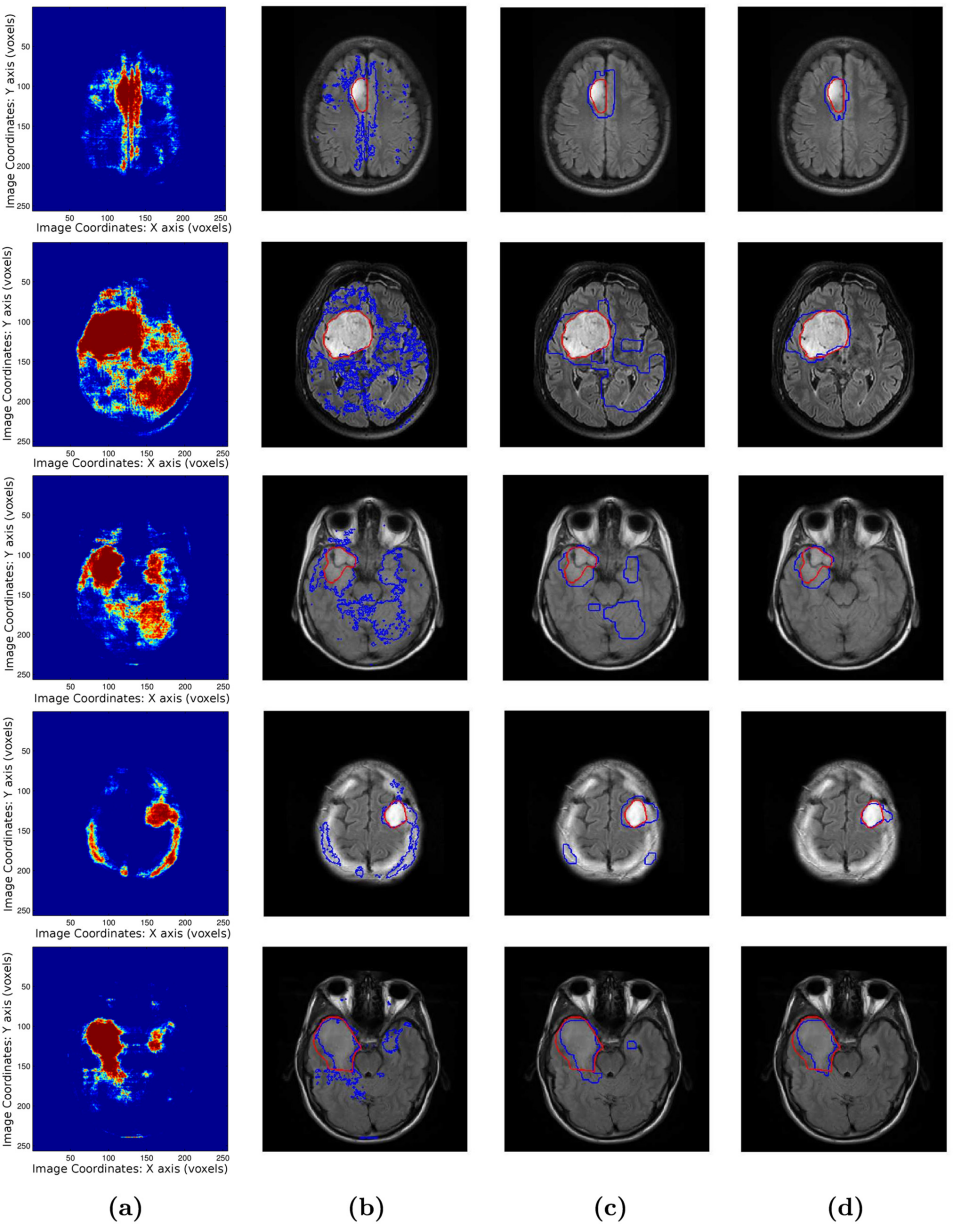
Visual Segmentation results. (a) boosting score, (b) Boosting classification (thresholding), (c) Pairwise MRF, (d) MRF with spatial prior.

Results are shown using boosting classification, boosting with pairwise regularization, and boosting with pairwise regularization and spatial prior. Table 2 shows the mean and median scores for the different configurations. Boxplots of the values are shown in Fig 7, while visual examples of segmentation results are illustrated in Fig 8. We observe an increase of the Dice score (+ 3%) as well as a strong decrease of the false positive rate (- 9%) with respect to the regularized method. The MAD score provides the most interesting evaluation since it is not biased by the size of the tumor (contrarily to the other measures). We can observe a strong improvement of the MAD score using the probabilistic atlas. A decrease of the true positive rate is observed, that is mostly associated to small and poorly detected tumors. In that case, the selected cluster may be inadequate and cause a decrease in the quality of the segmentation.

**Table 2:** Mean and median (in parentheses) values of the different scores and methods for tumor segmentation.

Method	Dice	True Positive	False Positive	MAD
Boosting	50 (55)%	77 (83)%	59 (58)%	22 (22) mm
Boosting regularized	66 (74)%	70 (78)%	29 (21)%	9.5 (6) mm
Our method	69 (77)%	66 (74)%	20 (12)%	6 (3.5) mm

### Correlation between Tumor Topography and Patient’s Age

**Fig 9:**
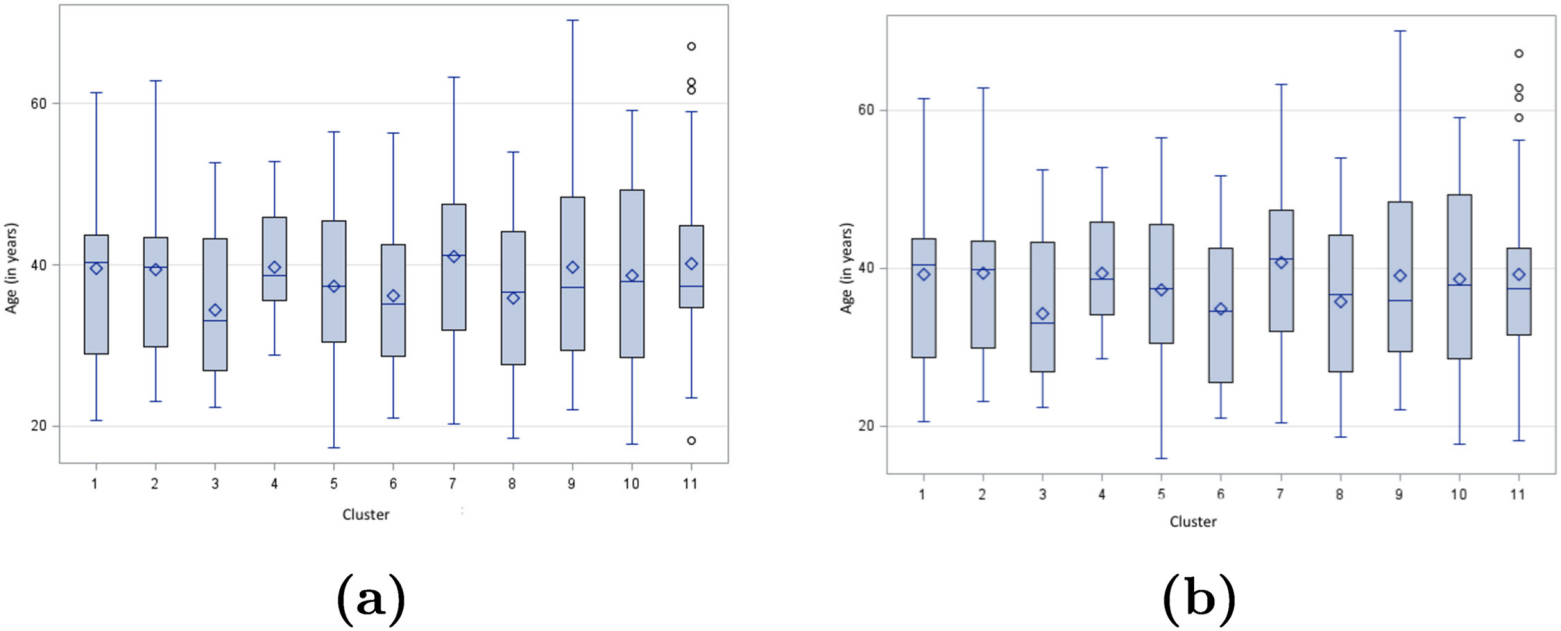
Patient’s age at the time of the first symptoms (a) and MRI diagnosis (b) for each cluster.

The ages at the time of the first symptoms and at the time of MRI diagnosis are described for each of the 11 clusters in Tables 3 and 4 and Fig 9 (box-plots). The analysis of variance found no significant difference in terms of age at the time of the first symptoms or age at the time of MRI diagnosis (p = 0.73 and p = 0.72, respectively). Nevertheless, patients whose MRI belongs to cluster 3 seem to be younger than the others, with a median age at 33,0 years at the time of the first symptoms and at 33.1 years at the time of the MRI diagnosis. When comparing this group of patients (n = 18) with all the others (n = 192), we found no significant difference as regards to age, with p = 0.11 for the age at the time of the first symptoms and p = 0.91 for age at the time of MRI diagnosis, respectively. However, it is likely that actual differences exist since the 95% confidence intervals for the difference between both mean ages were very unbalanced around the zero value: [−0.99; 9.48] for the age at the time of the first symptoms and [−0.74; 9.79] for the age at the time of the MRI diagnosis.

**Table 3:** Mean and median ages at the time of the first symptoms for each cluster.

		Age at the time of the first symptoms (in years)				
Cluster	Nsamples (%)	Mean	Standard Deviation	Median	Min	Max
1	9 (4.3)	39.1	12.2	40.4	20.5	61.4
2	17 (8.1)	39.3	10.7	39.8	23.1	62.8
3	18 (8.6)	34.2	8.6	33.0	22.3	52.4
4	13 (6.2)	39.4	7.5	38.6	28.6	52.7
5	19 (9.0)	37.2	10.3	37.3	15.9	56.5
6	12 (5.7)	34.7	10.8	34.5	21.0	51.7
7	25 (11.9)	40.7	10.0	41.2	20.3	63.2
8	19 (9.0)	35.6	10.5	36.6	18.5	53.9
9	30 (14.3)	39.0	12.6	35.8	22.0	70.0
10	19 (9.0)	38.6	11.2	37.9	17.7	59.1
11	29 (13.8)	39.1	12.1	37.3	18.2	67.1

**Table 4:** Mean and median ages at the time of MRI diagnosis for each cluster.

	Age at the time of MRI diagnosis (in years)				
Cluster	Mean	SD	Median	Min	Max
1	39.5	12.5	40.4	20.7	61.4
2	39.3	10.7	39.8	23.1	62.8
3	34.4	8.8	33.1	22.3	52.7
4	39.7	7.4	38.6	28.9	52.9
5	37.4	10.2	37.3	17.3	56.6
6	36.2	10.7	35.1	21.0	56.4
7	41.0	10.2	41.2	20.3	63.2
8	35.8	10.6	36.7	18.5	54.0
9	39.6	12.7	37.2	22.0	70.3
10	38.6	11.2	38.0	17.8	59.1
11	40.2	12.2	37.4	18.2	67.1

## Discussion

In this paper, we introduced a probabilistic atlas that identifies the preferential locations of DLGGs in the brain. This atlas is based on a homogeneous series of non-treated DLGG, which is, to our knowledge, the biggest series of “pure” DLGG. We analyzed MRIs only if they had been performed before the patient had received any oncological treatment. Because the MRIs in DICOM format and with FLAIR sequences were necessary for our analysis, we could not use the first MRI performed (i.e. the MRI at diagnosis) for some patients (n = 122). However for these patients the median delay between the first MRI and the MRI analyzed was short (125 days), and was over a year for 19 patients only. As DLGG are slowly growing tumors, this delay is unlikely to cause dramatic changes in the tumor volume or to bias the determination of the tumor center of gravity.

The atlas provides a precise description of the tumors locations and spatial extension in each cluster (prior probability maps and clusters positions). The insula is undoubtedly a predominant location for the DLGG as more than a third of the tumors are situated in this area (37% of the data). This result, as well as the quasi-total absence of tumors in or near the occipital and prefrontal lobes, is in accordance with what was observed in [17]. The proposed atlas and complete database are available for download at http://db-gliomas-gradeii.net/.

Several applications for this atlas are imaginable. The proposed atlas constitutes a robust method for correlations between DLGG topography and their characteristics. The patient’s age at diagnosis and first symptoms was considered in this paper, and a study with respect to the tumors’ molecular biology is currently ongoing.

We were not able to show any variability regarding both the age at the moment of the first symptoms or the age at MRI diagnosis according to the tumor location. Such variability has been recently suggested by Gerin et al, who identified two types of tumors among DLGGs, the first one occurring during teenage years with a very slow growth, and the second one occurring in early adulthood with a slow growth, [5]. In our series there is a trend towards a younger age for patients with a tumor belonging to the cluster 3 involving the SMA compared to the other locations, but it did not reach significance. However, it is likely that there is actually a difference with regard to the age since the 95% confidence intervals of the difference between mean ages of the “SMA group” and the “non-SMA” group, even though they contain the value zero, are unbalanced towards zero ([−0.99; 9.48]) for the age at the time of the first symptoms and [−0.74; 9.79] for at the time of MRI diagnosis, respectively). The small effective in the “SMA group” (n = 18) could partly explain our lack of statistic power. If such a difference does exist for DLGG involving the SMA, it would reinforce the fact that this area has specific biological, architectonic and functional properties, with particular interactions between glial cells and neurons that could, maybe, explain in parts earlier gliomatogenesis [17].

This study should be pursued with a larger database in order to establish whether those tendencies are confirmed. Furthermore, we have now designed a pipeline for correlating the position of the tumor with any parameter through the use of the probabilistic atlas. It is straightforward to extend the study to investigate the correlation of other parameters (molecular biology for instance) to the tumor location.

Furthermore, it would be interesting to couple this atlas with information obtained from functional imaging and study the relationship between tumors’ position and brain functions. This could enable to identify compensable regions and predict the future functional reorganization of the brain depending on the location of the tumor. All of this would be extremely useful for surgery planning. Eventually, the same method could also be applied to other kinds of lesions, perhaps by changing the distance measure for one that is more adapted to the type of lesion.

We presented in this paper an application as a spatial position prior for tumor detection and segmentation. We managed to efficiently detect and characterize (i.e. assign to a cluster) the tumors from a sizable dataset and despite some unclear boundaries and heterogeneous appearances. As stated in the introduction, DLGG have different behaviors depending on their location. We could be able to infer the evolution of the tumor depending on the cluster it is assigned to.

Obtaining the best possible tumor segmentation is not the main focus of this paper. The proposed method was used to highlight the clinical relevance of the probabilistic atlas. We therefore used simple unary costs (boosting) while much more elaborated approaches from the literature could have been considered. It is therefore difficult to compare our results with the current state of the art from the BRATS challenge [50]. On top of that, we are using a very different and challenging database (monomodal data, variable quality, low resolution) due to our clinical setting.

Segmentation can be harder for small tumors, that do not offer an important contrast and are sometimes even difficult to detect visually. The impact of the spatial prior on the segmentation quality is directly dependent on the accuracy of the cluster assignment as well as how well the tumor fits in the cluster. As stated above, 12% of the data could not be represented by any cluster and therefore should not be assigned to any. The segmentation results would most likely be increased by the introduction of a label identifying outlier tumors, possibly by identifying if the tumor is equally distant to two or more clusters.

Despite the fact that we are already working on a large data-set, the quality and precision of the graph as well as the clusters probability distributions (i.e. the prior probability maps), could benefit from an increase of the size of the data set. Currently, we evaluate the segmentation’s quality with respect to a manual segmentation by comparing the pixels detected as tumor to the pixels manually labeled as tumors. Manual segmentation is, unfortunately, a subjective approach and dependent on the operator [28]. Results could differ with another operator, especially in the case of DLGG where boundaries of the tumors can be very fuzzy.

The registration step also leaves room for improvement. First, the fact that all registrations are done towards an arbitrary reference pose introduces a bias in the atlas’ structure. We should get better results using population registration methods [51] that register all the images together without the use of a reference pose. Second, the use of an affine registration scheme maintains the different brains anatomy but lacks precision in the matching of the anatomical structures. Increased precision would be obtained by aligning all volumes through a deformable registration scheme that takes into account the missing correspondences induced by the presence of the tumor [33]. Due to the systematic processing of a potentially large scale database, deformable registration methods have to be made fully reliable before being used in our context.
